# Resource Utilization and Costs of Care prior to ART Initiation for Pediatric Patients in Zambia

**DOI:** 10.1155/2014/235483

**Published:** 2014-03-10

**Authors:** Hari S. Iyer, Callie A. Scott, Deophine Lembela Bwalya, Gesine Meyer-Rath, Crispin Moyo, Carolyn Bolton Moore, Bruce A. Larson, Sydney Rosen

**Affiliations:** ^1^Zambia Center for Applied Health Research and Development, 10100 Lusaka, Zambia; ^2^Center for Global Health and Development, Boston University, Boston, MA 02118, USA; ^3^Health Economics and Epidemiology Research Office, Wits Health Consortium, Department of Clinical Medicine, School of Internal Medicine, Faculty of Health Sciences, University of the Witwatersrand, Johannesburg 2198, South Africa; ^4^Zambian Ministry of Health, 10100 Lusaka, Zambia; ^5^Department of Obstetrics and Gynecology, University of North Carolina at Chapel Hill, Chapel Hill, NC 27514, USA; ^6^Centre for Infectious Disease Research in Zambia, 101000 Lusaka, Zambia; ^7^Department of International Health, School of Public Health, Boston University, Boston, MA 02118, USA

## Abstract

*Objective*. We estimated time to initiation, outpatient resource use, and costs of outpatient care during the 6 months prior to ART initiation for HIV-infected pediatric patients in Zambia. *Methods*. We enrolled 1,102 children who initiated ART at <15 years of age between 2006 and 2011 at 5 study sites. Of these, 832 initiated ART ≤6 months after first presenting to care at the study sites. Data on time in care and resources utilized during the 6 months prior to ART initiation were extracted from patient medical records. Costs were estimated from the provider's perspective and are reported in 2011 USD. *Results*. For the patients who initiated ART ≤6 months after presenting to care, median age at presentation to care was 3.9 years; median CD4 percentage was 13%. Median time to ART initiation was 26 days. Patients made, on average, 2.38 clinic visits prior to ART initiation and received 0.81 CD4 tests, 0.74 full blood count tests, and 0.49 blood chemistry tests. The mean cost of pre-ART care was $20 per patient. *Conclusions*. Zambian pediatric patients initiating ART ≤6 months after presenting to care do so quickly, utilize fewer resources than mandated by national guidelines, and accrue low costs.

## 1. Introduction

Of the roughly 170,000 children living with HIV in Zambia in 2011, only an estimated 30,187 were receiving antiretroviral therapy (ART) in that year [1, 2]. Due to the difficulty of following pediatric patients from diagnosis of HIV infection to ART initiation, little information is available about the care provided to HIV-infected children prior to ART initiation [3–7]. Using cohort data collected for a larger study of the costs of pediatric ART in Zambia, we describe time to ART initiation, outpatient resource use, and costs of outpatient care during the pre-ART period for pediatric patients initiating ART within six months of presenting to care.

## 2. Materials and Methods

### 2.1. Analytic Overview

We enrolled a retrospective cohort of HIV-infected children who initiated ART at five treatment sites in Zambia between 2006 and 2011. We collected patient-level data on resource utilization during the six months prior to ART initiation from outpatient medical records and site- and country-level data on unit costs. We estimated time to ART initiation as well as the average quantity of outpatient resources utilized and the average outpatient cost per patient during the six months prior to ART initiation. Costs were calculated from the provider's perspective in 2011 US dollars.

### 2.2. Study Sites

Five study sites out of nearly 400 clinics providing ART in Zambia as of 2010 were purposively (not randomly) chosen to represent different models of pediatric ART delivery in Zambia. These have been described previously [8]. They included a health center in Lusaka Province, a second-level general hospital in Western Province, a second-level general hospital in Copperbelt Province, a second-level mission hospital in Southern Province, and a first-level district hospital in Southern Province.

### 2.3. Study Population

We enrolled a consecutive sample of 1,102 pediatric patients initiating ART at the study sites between 2006 and 2011 using clinic registers. Eligible patients were <15 years of age at the time of ART initiation and were not known to have been transferred to another clinic during the study follow-up period. Of these 1,102 eligible patients, 151 received no care at the study sites prior to ART initiation and were not included in this analysis of pre-ART care. Among the 951 who received any care at the study sites prior to ART initiation, 832 (88%) initiated ART within six months of presenting to care and 119 (12%) initiated ART more than six months after presenting to care. In this paper, we report on time to ART initiation and average resource utilization and costs during the six months prior to ART initiation for the 832 patients who initiated ART within six months of presenting to care at the study sites.

Patients in the sample were eligible to initiate ART according to Zambian national guidelines prevailing during the study period [9]. All children classified as WHO clinical stage 3 or 4 were eligible to initiate ART. Children classified as WHO clinical stage 1 or 2 could initiate ART based on age-specific CD4 or total lymphocyte count criteria.

### 2.4. Ethics Statement

The Boston University Medical Center Institutional Review Board and the University of Zambia Research Ethics Committee provided ethical approval of the study (protocol numbers H-28104 and 008-04-09). A waiver of informed consent was granted by both committees because the study was a retrospective review of routinely collected information from patient medical records.

### 2.5. Unit Cost Estimates

We used previously published methods to estimate unit costs in 2011 US dollars for outpatient resources utilized by study patients (fixed resources, laboratory tests, and clinic visits) [8]. Fixed resources included buildings, equipment, and support staff in the ART clinic. For buildings and equipment, we estimated upfront investment costs using a replacement cost approach and then we used a 3% discount rate and estimated working life (50 years for buildings, 5 years for equipment) to calculate an annualized cost [10]. The cost of support staff was calculated using 2011 salaries and allowances. A fixed cost per patient-year in care was calculated by summing all fixed costs at each study site and then dividing by the total number of active patients (pre-ART and on-ART) at the site during each year.

Laboratory test costs were estimated by summing the unit costs for reagents, consumables, equipment, labor, and space. For reagents and consumables, unit costs were estimated from standard Zambian Ministry of Health per package costs [11]. Equipment, labor, and space costs per laboratory test were estimated by dividing the annualized costs of laboratory equipment, labor, and space by the total number of each type of laboratory test performed at the site per year. The cost per clinic visit with each type of provider was estimated by dividing the annual cost of staff time, based on 2011 salaries and allowances, for each provider type by the number of patient consultations with that provider type per year.

### 2.6. Data Analysis

We calculated the total quantity of each resource utilized by each patient during their time in pre-ART care and then calculated total pre-ART costs for each patient by multiplying the unit cost for each resource by the total quantity utilized. We calculated average resource utilization and costs by dividing total resource utilization and costs for all patients by the total number of patients in the cohort.

We stratified results by the number of clinic visits completed during the pre-ART period (≤2 visits, >2 visits) to compare resource utilization for patients who received more or less care prior to ART initiation. Differences in means were calculated using an independent two-sided *t*-test with unequal variances. Differences in medians were calculated using the Wilcoxon rank sum test. A *P* value of less than 0.05 was regarded as significant.

## 3. Results and Discussion

### 3.1. Cohort Characteristics

Median age upon presentation to care was 3.9 years and median CD4 percentage was 13%; 51% of patients were female (Table 1). Compared to patients with >2 visits to the study sites during the six months prior to ART initiation, patients with ≤2 visits were slightly older (4.1 years versus 3.6 years) and had a slightly lower median CD4 percentage (13% versus 14%) upon presentation to care.

### 3.2. Resource Utilization

Patients spent a median of 26 days in care prior to ART initiation (Table 2). On average, patients made 2.38 clinic visits to the study sites and received 0.81 CD4 tests, 0.74 full blood count tests, and 0.49 blood chemistry tests during the six months prior to ART initiation.

Thirty-nine percent of patients had >2 clinic visits at the study sites during the six months prior to ART initiation. Patients with >2 visits spent significantly more days in care (43 versus 17, *P* < 0.0001) and had significantly more CD4 tests (1.1 versus 0.7, *P* < 0.0001), full blood count tests (1.0 versus 0.6, *P* < 0.0001), and clinic visits (3.7 versus 1.6, *P* < 0.0001) than patients with ≤2 visits.

### 3.3. Costs of Care

The average cost of care received at the study sites over the six-month period prior to ART initiation was $20. The cost for patients with >2 visits averaged $25 while the cost for patients with ≤2 visits averaged $17.

## 4. Discussion

We estimated time to ART initiation, the average quantity of outpatient resources utilized, and average outpatient costs during the six months prior to ART initiation for a cohort of 832 pediatric patients who initiated ART within six months of presenting to care at five sites in Zambia between 2006 and 2011. While we only have reported CD4 percentage at presentation for 231 patients in our sample, their median CD4 percentage of 13% suggests that many of these patients presented late to care. Pediatric patients in our sample spent a median of just under a month in care prior to ART initiation. They used few resources (mean 2.38 clinic visits, 0.81 CD4 tests, 0.74 full blood count tests, and 0.49 blood chemistry tests) and incurred an average cost of only $20 for care provided during the six months prior to ART initiation.

Our finding that pediatric patients who initiated ART within six months of presenting to care presented with a median CD4 percentage of 13% and initiated ART soon after presenting to care suggests that many of these patients might have been ART eligible upon presentation to care or at the very least became eligible very soon afterwards. For HIV-infected children presenting to care late in the course of disease progression, a median of 26 days between presentation to care and ART initiation may be too long given the high risk of mortality prior to ART initiation [6, 12, 13].

Our findings also suggest that pediatric patients utilized fewer resources than expected if they were receiving fully guideline-concordant care prior to ART initiation. Zambian national guidelines for pediatric ART recommend that patients receive at least one CD4 test, one full blood count test, and one blood chemistry test prior to ART initiation, more than the average of 0.81 CD4 tests, 0.74 full blood count tests, and 0.49 blood chemistry tests for children in our sample [9].

Few studies have documented pre-ART resource use and costs of care among pediatric patients in southern Africa. Desmonde et al. found that pediatric pre-ART patients in Côte d'Ivoire who went on to initiate ART spent a median of 27 days in care prior to ART initiation, similar to our finding of 26 days [14].

The average cost per patient of $20 for the median 26 days spent in care prior to ART initiation in this study is also comparable to previous estimates of pre-ART costs and to on-ART costs excluding ARV drugs. We previously estimated an annual cost of $131 per pediatric ART patient remaining in care one year after initiating ART in Zambia if ARV drugs were excluded, equivalent to an average monthly cost of $11 [8]. Bratt et al. estimated a cost per ART initiation visit of $27 and a cost per ART follow-up visit of $10, after excluding ARV drug costs, for adults and children at ART clinics in Zambia [15].

Our study had several limitations, largely stemming from the difficulty of utilizing routinely collected medical record data from health facilities. Our sample only included patients who ultimately initiated ART at five sites that were not selected randomly; so our average costs and time spent in pre-ART care cannot be generalized to the country as a whole. While we have complete data on care received prior to ART initiation at the study sites for 983 out of 1,102 patients (151 who received no pre-ART care and 832 who received >0 and ≤6 months of pre-ART care), we do not have similar information for the 119 of 1,102 patients who received >6 months of pre-ART care at the study sites. We therefore can neither estimate the full cost of pre-ART care nor describe retention in pre-ART care. We also excluded the costs of drugs, inpatient care, or other care received outside of the study site, costs incurred by the patient, and costs for program management above the facility level, all of which would increase the average cost per patient in pre-ART care. For children who present late to care, the costs of treatment for additional opportunistic infections and comorbidities could be considerable. Finally, we do not comment on the variability in demographics and costs across sites, although these differences are discussed elsewhere [8].

## 5. Conclusions

In summary, our findings suggest that pediatric patients in Zambia spend about a month in pre-ART care, use fewer resources than mandated by national guidelines, and accrue relatively low costs during the six months prior to ART initiation. Further research that examines the benefits of pre-ART care for patient outcomes and estimates attrition between initial presentation and ART initiation would help local and international policymakers improve pediatric HIV care.

## Figures and Tables

**Table 1:** Cohort characteristics for an analysis of time to initiation, resource utilization, and costs of care during the 6 months prior to treatment initiation for pediatric ART patients in Zambia.

	Patients with ≤2 clinic visits prior to ART initiation *N* = 511	Patients with >2 clinic visits prior to ART initiation *N* = 321	All patients *N* = 832
Age in years at presentation to care at study site, median [IQR]	4.1 [1.7–8.1]	3.6 [1.5–7.3]	3.9 [1.7–7.8]
Female, *n* (%)*	229 (53)	114 (47)	343 (51)
CD4 percentage at presentation to care at study site, median [IQR]**	13 [8–18]	14 [10–19]	13 [9–19]

ART: antiretroviral therapy; IQR: interquartile range.

*We only report gender information for the 679 patients for whom this information was available.

**CD4 percentage at presentation care at the study site was the earliest reported CD4 percentage within one month of presentation to care at the study site and prior to ART initiation. CD4 percentages were only available for 231 patients.

**Table 2:** Resource utilization and costs of care during the 6 months prior to treatment initiation for pediatric ART patients in Zambia.

	Patients with ≤2 clinic visits prior to ART initiation	Patients with >2 clinic visits prior to ART initiation	*P* value	All patients
*N* (%)	511 (61)	321 (39)		832 (100)
Days to ART initiation, median [IQR]	17 [10–29]	43 [28–83]	<0.0001	26 [14–48]
Resource utilization				
Clinic visits, mean (95% CI)	1.56 (1.51–1.60)	3.69 (3.56–3.81)	<0.0001	2.38 (2.29–2.47)
CD4 tests, mean (95% CI)	0.67 (0.62–0.71)	1.05 (0.98–1.11)	<0.0001	0.81 (0.77–0.85)
Full blood count tests, mean (95% CI)	0.56 (0.51–0.61)	1.03 (0.96–1.11)	<0.0001	0.74 (0.70–0.79)
Blood chemistry tests, mean (95% CI)*	0.48 (0.44–0.52)	0.49 (0.44–0.55)	0.80	0.49 (0.45–0.52)
Cost per patient in 2011 USD, mean (95% CI)	17 (16–18)	25 (23–26)	<0.0001	20 (19–21)

ART: antiretroviral therapy; USD: United States dollar.

*A blood chemistry test could include any combination of the following tests: alanine aminotransferase, aspartate aminotransferase, creatinine, glucose, protein, urea, total bilirubin, and direct bilirubin tests.

## References

[1] Government of the Republic of Zambia, Ministry of Health and the National AIDS Council (2012). Zambia country report: Monitoring the declaration of commitment on HIV and AIDS and the universal access. *Biennial Report*.

[2] UNAIDS Zambia HIV and AIDS estimates. http://www.unaids.org/en/regionscountries/countries/zambia/.

[3] World Health Organization Global HIV/AIDS response: epidemic update and health sector progress towards universal access: progress report. http://whqlibdoc.who.int/publications/2011/9789241502986_eng.pdf.

[4] Orne-Gliemann J, Becquet R, Ekouevi DK, Leroy V, Perez F, Dabis F (2008). Children and HIV/AIDS: from research to policy and action in resource-limited settings. *AIDS*.

[5] Rosen S, Fox MP (2011). Retention in HIV care between testing and treatment in sub-Saharan Africa: a systematic review. *PLoS Medicine*.

[6] Anaky M-F, Duvignac J, Wemin L (2010). Scaling up antiretroviral therapy for HIV-infected children in Côte d’Ivoire: determinants of survival and loss to programme. *Bulletin of the World Health Organization*.

[7] Raguenaud M-E, Isaakidis P, Zachariah R (2009). Excellent outcomes among HIV+ children on ART, but unacceptably high pre-ART mortality and losses to follow-up: a cohort study from Cambodia. *BMC Pediatrics*.

[8] Scott CA, Iyer H, Lembela Bwalya D (2013). Retention in care and outpatient costs for children receiving antiretroviral therapy in Zambia: a retrospective cohort analysis. *PLoS ONE*.

[9] Government of the Republic of Zambia, Ministry of Health Zambian guidelines for antiretroviral therapy of HIV infection in infants and children: towards universal access. http://www.who.int/hiv/amds/zambia_paediatric_guidelines_2007.pdf.

[10] Weinstein MC, Siegel JE, Gold MR, Kamlet MS, Russell LB (1996). Recommendations of the panel on cost-effectiveness in health and medicine. *Journal of the American Medical Association*.

[11] Limited MS (2010). *Medical Stores Limited: 2010 Catalogue*.

[12] Bolton-Moore C, Mubiana-Mbewe M, Cantrell RA (2007). Clinical outcomes and CD4 cell response in children receiving antiretroviral therapy at primary health care facilities in Zambia. *Journal of the American Medical Association*.

[13] Walker AS, Mulenga V, Sinyinza F (2006). Determinants of survival without antiretroviral therapy after infancy in HIV-1-infected Zambian children in the CHAP trial. *Journal of Acquired Immune Deficiency Syndromes*.

[14] Desmonde S, Coffie P, Aka E (2011). Severe morbidity and mortality in untreated HIV-infected children in a paediatric care programme in Abidjan, Côte d’Ivoire, 2004–2009. *BMC Infectious Diseases*.

[15] Bratt JH, Torpey K, Kabaso M, Gondwe Y (2011). Costs of HIV/AIDS outpatient services delivered through Zambian public health facilities. *Tropical Medicine and International Health*.

